# Comments on “Evaluation of the Accuracy of Mammography, Ultrasound and Magnetic Resonance Imaging in Suspect Breast Lesions”

**DOI:** 10.6061/clinics/2020/e2338

**Published:** 2020-11-10

**Authors:** Yongyu An

**Affiliations:** Department of Radiology, Tongde Hospital of Zhejiang Province, Zhejiang Province, China

Dear Editor:

I read the article (1) “Evaluation of the accuracy of mammography, ultrasound and magnetic resonance imaging in suspect breast lesions” (Pereira et al.) with great interest; however, I am left confused by the study and believe some issues must be discussed.

The aim of this study was to compare the diagnostic performance of three imaging methods for breast cancer (1). However, all patients showed a suspect breast lesion on evaluation by at least one of the methods. Based on the Breast Imaging-Reporting and Data System (BI-RADS), biopsy or surgical resection was recommended; however, the positive predictive value for BI-RADS categories 4 and 5 varies depending on the imaging modality. For instance, almost 90% of the suspect lesions detected by ultrasound eventually show negative results, leading to unnecessary biopsies (2). In this setting, the role of other imaging modalities is to evaluate whether a suspected lesion is truly malignant. Hence, magnetic resonance imaging (MRI) is the superior modality as it shows high sensitivity for breast cancer detection and has a high negative predictive value for the exclusion of malignancy (3-5), as shown in the study by Pereira at al. (1). Furthermore, several studies have suggested that MRI could be used as a problem-solving tool in cases of suspicious clinical and radiological findings (6-8).

Finally, I do not agree with the statement that MRI has low specificity owing to high breast density. Breast density has a vital impact on the interpretation of mammography findings, but not on that of MRI findings. On dynamic contrast-enhanced MRI, both morphology and kinetics are key for the diagnosis of breast lesions, although there is considerable overlap between signs of benign and malignant lesions, resulting in false-positive findings. Several studies have revealed that diffusion-weighted imaging can improve the specificity of breast MRI and help avoid unnecessary biopsies (9,10). The specificity shown in this study was low (1); hence, I wonder whether diffusion-weighted imaging was integrated into breast MRI. I would be interested in receiving responses to my comments.
